# Gateway Reflex and Mechanotransduction

**DOI:** 10.3389/fimmu.2021.780451

**Published:** 2021-12-22

**Authors:** Shiina Matsuyama, Yuki Tanaka, Rie Hasebe, Shintaro Hojyo, Masaaki Murakami

**Affiliations:** ^1^ Molecular Psychoimmunology, Institute for Genetic Medicine, Graduate School of Medicine, Hokkaido University, Sapporo, Japan; ^2^ Group of Quantum Immunology, Institute for Quantum Life Science, National Institute for Quantum and Radiological Science and Technology (QST), Chiba, Japan; ^3^ Division of Neurommunology, National Institute for Physiological Sciences, Okazaki, Japan

**Keywords:** gateway reflex, mechanotransduction, inflammation, tissue specific autoimmune diseases, CD4+ T cells

## Abstract

The gateway reflex explains how autoreactive CD4+ T cells cause inflammation in tissues that have blood-barriers, such as the central nervous system and retina. It depends on neural activations in response to specific external stimuli, such as gravity, pain, stress, and light, which lead to the secretion of noradrenaline at specific vessels in the tissues. Noradrenaline activates NFkB at these vessels, followed by an increase of chemokine expression as well as a reduction of tight junction molecules to accumulate autoreactive CD4+ T cells, which breach blood-barriers. Transient receptor potential vanilloid 1 (TRPV1) molecules on sensory neurons are critical for the gateway reflex, indicating the importance of mechano-sensing. In this review, we overview the gateway reflex with a special interest in mechanosensory transduction (mechanotransduction).

## Mechano-Sensing Receptors

Mechano-sensing is a general cellular phenomenon in which changes in membrane tension and the cytoskeletal structure induce intracellular signal transduction in response to an extracellular mechanical force (1). Many receptors are sensitive to mechanical forces. The list includes Twik-related K^+^ channel (TREK) and Twik-related arachidonic acid-stimulated K^+^ (TRAAK), both of which being two-pore domain K+ (K2p) channels, Piezo1/2, TMEM63/OSCA, and transmembrane channel-like (TMC)1 and TMC2. These mechanosensory ion channels (MSCs) all sense extracellular stimulations, such as pressure, gravity, acceleration, sound waves, tension, fluid flow, pain, light, temperature, and blood pressure (2–6). In addition, cells adhere to neighboring cells and to the extracellular matrix *via* transmembrane receptors of the cadherin (cell-to-cell) and integrin (cell-to-substrate) families. These molecules are related to cell adhesion but are also mechano-sensitive (7–9), suggesting they are mechano-sensing receptors in the broad sense of the term. The extracellular stimuli are converted by MSCs and adhesion molecules into intracellular signals *via* alterations in the intracellular ion concentration or intracellular cytoskeletal status in various cells including sensory neurons and endothelial cells (10).

## MSCs and Adhesion-Related Molecules in Mechanotransduction

The above MSCs have been categorized as depolarizing cationic non-selective channels, such as Piezo1/2, and hyperpolarizing K+-selective stretch-activated channels, such as TREK/TRAAK K2p channels, TMEM63/OSCA, and TMC1/2. In mammalian cells, after the binding of a ligand or change in membrane tension around them, these channels change their structurers to shift the balance of the intracellular/extracellular ion concentration (2–5, 11). Piezo1/2 increase intracellular Ca^2+^, while the other channels decrease intracellular K^+^, thus augmenting the effects of intracellular Ca^2+^. Degenerin channels (DEG), epithelial sodium channels (ENaC), acid-sensing ion channels (ASICS), and transient receptor potentials (TRP) channels are other MSCs. Recent electron cryo-microscopy structure studies for TRPs have revealed that a helical spring structure displaces intracellular cytoskeleton molecules to open the channels upon mechanical stress (11–13).

**Figure 1:**
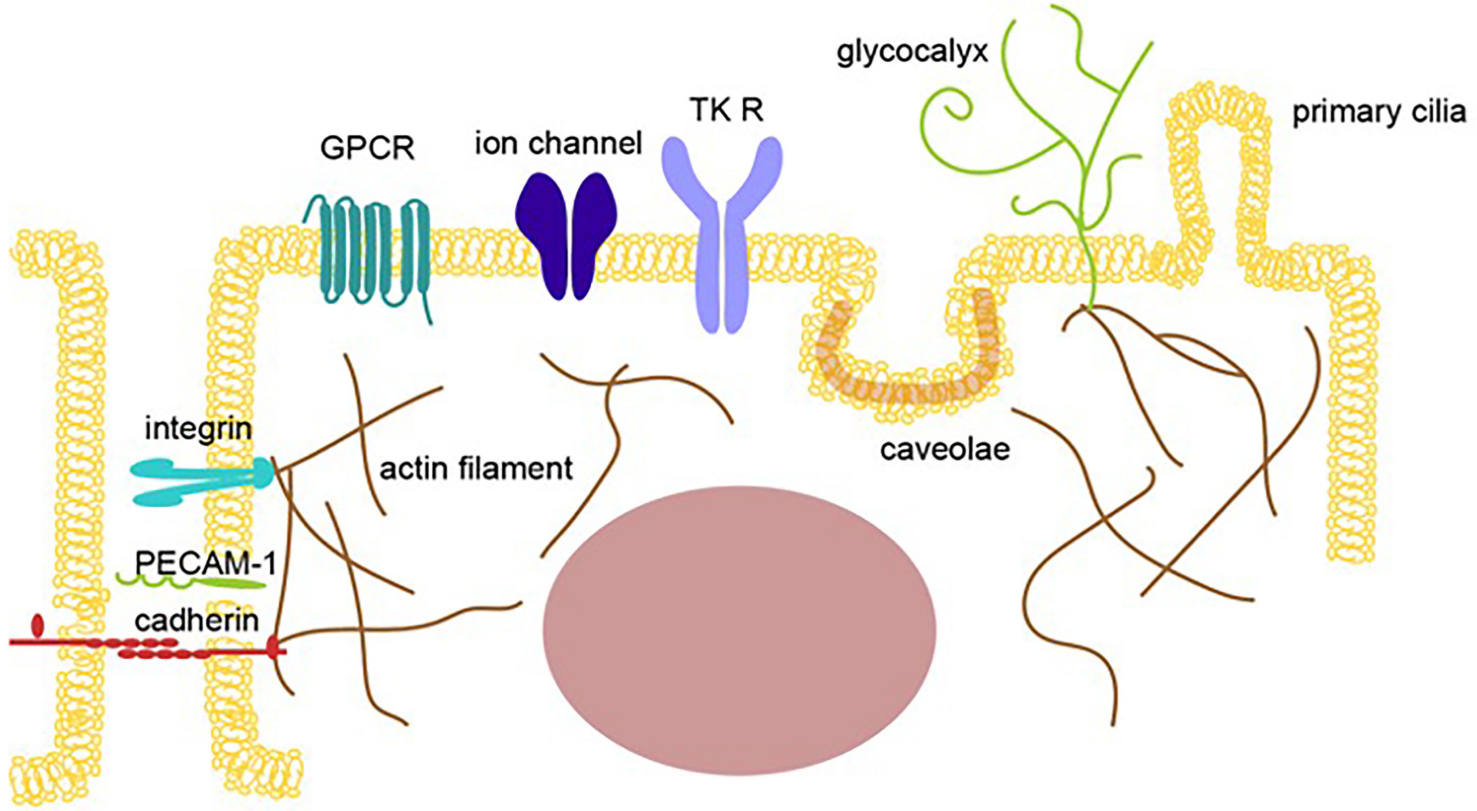
Mechano-sensing receptors in endothelial cells. An illustration showing multiple mechano-sensing receptors, including mechanosensory ion channels and adhesion molecules, that can respond to extracellular stimulations, such as pressure, gravity, acceleration, sound, tension, fluid flow, pain, light, temperature, and blood pressure, to induce mechanotransduction in endothelial cells. The sensors shown include tyrosine kinase receptors (TK R), G protein-coupled receptors, ion channels, junction proteins, integrins, the cell membrane (caveolae), and glycocalyx.

Endothelial cells in the blood vessels contact each other as a monolayer to pass essential factors, potential energy, and sometimes immune cells for the surveillance of tumor development and cells infected by pathogens from the blood to the tissues. Endothelial cells are constantly enduring mechanical stress from the blood flow, which is detected by transmembrane proteins, such as tyrosine kinase receptors, G protein-coupled receptors (GPCR), integrins, and cadherins, membrane structures, such as caveolae and primary cilia, glycocalyx, and cytoskeleton proteins, such as actin and tubulin, all of which transduce a mechanical signal to intracellular signals that cause transcriptional alterations to change the cellular status of endothelial cells (14–18)(**Figure 1**).

## The Gateway Reflex

Endothelial cells in the central nervous system (CNS) represent a specific vessel structure named the blood-brain barrier (BBB), which prevents peripheral immune cells and other factors including immunoglobulins from infiltrating the CNS from the blood. The BBB is important for maintaining the CNS environment and depends on many of the mechano-sensing molecules described above. However, observations of neuroinflammation due to an accumulation of immune cells in many diseases, including autoimmune diseases, schizophrenia, and Alzheimer’s disease, suggest the BBB can be compromised (19–24). To migrate past the BBB and into the CNS, peripheral immune cells particularly activated ones rely on adhesion molecules as well as chemokine molecules expressed by endothelial cells to affect mechanosensory pathways (25–29), because some signaling through adhesion molecules and chemokines directly increase molecules related to the mechanosensory pathways (25, 26, 30–32) and cytokines expressed from the immune cells accumulated also indirectly affect mechanosensory pathways (26–29). Because immune cells interact with endothelial cells in the BBB and affect the BBB structure and function, the status of the BBB is mechano-sensitive (30).

## Gravity and Electric Gateway Reflexes

Space experiments have shown that gravity affects bone and muscle density (31, 32) and indicate that the body transduces gravity into mechanical signaling. We demonstrated that gravity regulates tissue-specific CNS inflammation by showing that autoreactive CD4+ T cells invade the specific sites of the CNS in experimental autoimmune encephalomyelitis (EAE), an animal model of multiple sclerosis (33). EAE mice are an invaluable tool for studying the pathogenesis of the BBB and the accumulation of immune cells in the CNS. One of the most popular murine models is EAE induced by a myelin-derived antigen, myelin oligodendrocyte glycoprotein 35-55 (MOG35–55), which is given emulsified in complete Freund’s adjuvant (CFA) as a subcutaneous injection (34–37). The development of encephalomyelitis is observed as an ascending paralysis that begins with a drooping tail and progresses to paralysis of the lower limbs. The adoptive transfer of MOG-specific CD4+ T cells (pathogenic CD4+ T cells) from donor mice that were actively immunized by the MOG peptide with CFA to naïve recipient mice can also induce EAE. This adoptive transfer EAE model allows study of the autoimmune CNS inflammation induced specifically by pathogenic CD4+ T cells (38).

We found that in EAE mice transferred with pathogenic CD4+ T cells, the cells accumulated in the dorsal vascular sites of the fifth lumbar (L5) cord. Dorsal vascular endothelial cells of the L5 cord in EAE mice show an inflammation status including the expression of chemokines and growth factors followed by the accumulation of immune cells and proliferation of tissue nonimmune cells due to NFκB signaling. Pathogenic CD4+ T cells, but mainly Th17 ones, accumulate in the vascular endothelium and flow into the CNS using CCL20 as a chemotactic factor. After pathogenic CD4+ T cell accumulates, the BBB is breached due to various cytokines including NFκB and STAT stimulators from pathogenic CD4+ T cells, resulting in the accumulation of various immune cells from the blood to the L5 cord. This accumulation increases mechanical stress because of the increased cell density.

In a later experiment, we focused on sensory nerve input from the soleus muscles. The main anti-gravity muscles are the soleus muscles in both mice and human, and the sensory pathway from soleus muscles connects to the L5 dorsal root ganglion (DRG), which is the largest DRG and is activated by anti-gravity responses (33). Indeed, when the same experiment was performed on mice with their tails suspended to model a microgravity environment on the hind legs, CNS inflammation was induced in the cervical vessels but not the L5 vessels. Furthermore, electrically stimulating the soleus muscles, which mimics gravity stimulation, induced CCL20 at the L5 vessels. Moreover, electrically stimulating the quadriceps, whose afferent sensory nerves come from the L3 DRG, and the epitrochlearis/triceps brachii, whose afferent nerves come from between the fifth cervical and fifth thoracic DRG, caused CCL20 expression to increase in the L3 vessels and between the fifth cervical and fifth thoracic vessels, respectively.

**Figure 2:**
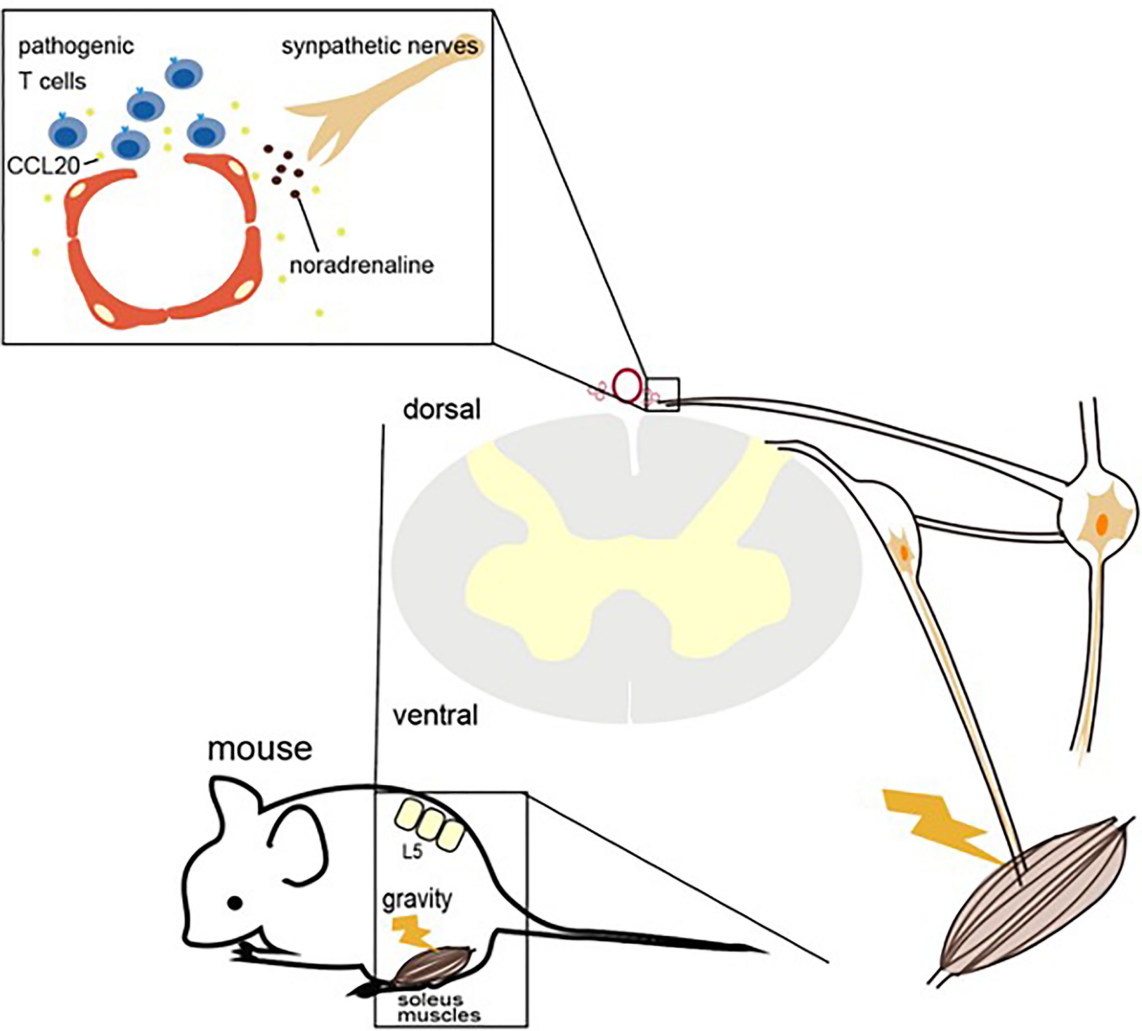
Light gateway reflex. The light gateway reflex. Photopic light stimulates a high expression of noradrenaline and adrenaline to downregulate a1A-adrenoceptor (a _1A_AR) expression on the retinal vessels in mice with autoreactive T cells against photoreceptors. The downregulation of a_1A_AR inhibits the noradrenaline-mediated activation of NFκB and STAT3, suppressing the IL-6 amplifier and retinal inflammation.

Sympathetic ganglions of the L5 level, but not other levels, were activated by gravity responses in the soleus muscles, and treatment with the norepinephrine antagonist atenolol significantly suppressed CCL20 mRNA expression, NFκB activation, and pathogenic CD4+ T cell accumulation around the L5 vessels and abrogated EAE development. These experiments indicate that sensory nerves in the soleus muscles that receive a gravity stimulus activate the sympathetic pathway at the L5 level, resulting in the production of noradrenaline, which upregulates CCL20 expression at the L5 dorsal vessels, although a detail crosstalk between sensory-sympatheic pathway in the L5 level has not been demonstrated. The resulting chemokine expression triggered inflammation around the vascular endothelium in the L5 cord, causing the accumulation of pathogenic CD4+ T cells from the blood and the development of neuroinflammation. These mechanisms, by which the nervous system regulates CNS inflammation in response to gravity stimulation or electric stimulation *via* the alteration of specific vessels, have been named the gravity gateway reflex and electric gateway reflex, respectively^　^(**Figure 2**) (33).

Gravity maintains the appropriate body state under healthy conditions. However, depending on the immune status of the individual, including the numbers of autoreactive T cells in the blood, it may also allow immune cells to invade the CNS and initiate autoimmune disease development *via* the gateway reflex. The receptors of sensory nerves that perceive gravity stimuli are still unclear, and the mechanosensory mechanism by which gravity stimuli activate the sensory nerves needs further investigation during both the gravity gateway reflex and electric gateway reflex.

## Pain Gateway Reflex

Two-thirds of patients with multiple sclerosis and particularly those with a relapse-remittent type will experience flare-ups (39). In contrast, in EAE mice transfected with pathogenic CD4+ T cells, the symptoms disappear within 2-3 weeks. Normally, EAE mice in remission show no relapse for more than 300 days after the transfer, but if pain stimulation is added, the symptoms relapse (40). The mouse trigeminal nerve is composed mainly of sensory nerve (41). Trigeminal neuropathic pain causes activation of the anterior cingulate cortex (ACC) and sensory neurons in the thalamus (42). A positive correlation has been reported between ACC activity, pain-induced sympathetic vasoconstrictor reflexes, and sympathetic responses to pain in humans, suggesting a functional link between the ACC, central sympathetic pathways, and pain experience (43). In remission EAE mice, trigeminal nerve ligation caused a relapse of the EAE symptoms, but a sham-operation did not. Capsaicin stimulation also induced the relapse, indicating that the pain-sensing TPRV1 channel activates sensory nerves and causes ACC activation. To confirm this hypothesis, pain stimulation was performed in TRPV1 knockout mice, but no relapse was observed. TRP channels are MSCs but also activated by heat, which alters the membrane tension and the status of intracellular cytoskeletal molecules (13). These results suggested that not only pain but also mechanical stimulations, such as compression, can induce the relapse *via* a similar neural pathway.

Mechanistically, pain sensation caused an accumulation of MHC2+CD11b+ myeloid cells at the ventral vascular blood vessels of the L5 cord, which triggered the accumulation of pathogenic CD4+ T cells in the blood and ultimately the infiltration of other immune cells into the spinal cord after breaching the BBB there. MHC class II+CD11b+ cells in EAE-recovered mice expressed not only MHC class II molecules but also co-stimulatory molecules, such as CD80, CD86, and intercellular adhesion molecule-1 (ICAM-1), which is a ligand of integrins and a mechano-sensing receptor. Importantly, MHC class II+CD11b+ cells have the ability to stimulate pathogenic CD4+ T cells without the addition of MOG peptide, suggesting they presented self-antigen peptides that stimulated pathogenic CD4+ T cells. We found that MHC2+CD11b+ cells increased the expression of CX3CR1 and its ligand CX3CL1 from L5 vessels in response to sympathetic noradrenaline. When CX3CL1 was inhibited with a neutralizing antibody, the relapse symptoms caused by pain stimulation were suppressed. Moreover, the inhibition of sympathetic nerve function by a β1-adrenagic receptor antagonist, atenolol, or the sympathectomy regent 6-hydroxydopamine (6-OHDA) suppressed the upregulated CX3CL1 expression and relapse, suggesting that not only the TRPV1+ sensory pathway but also the sympathetic pathway is critical for the pain gateway reflex (40).

**Figure 3:**
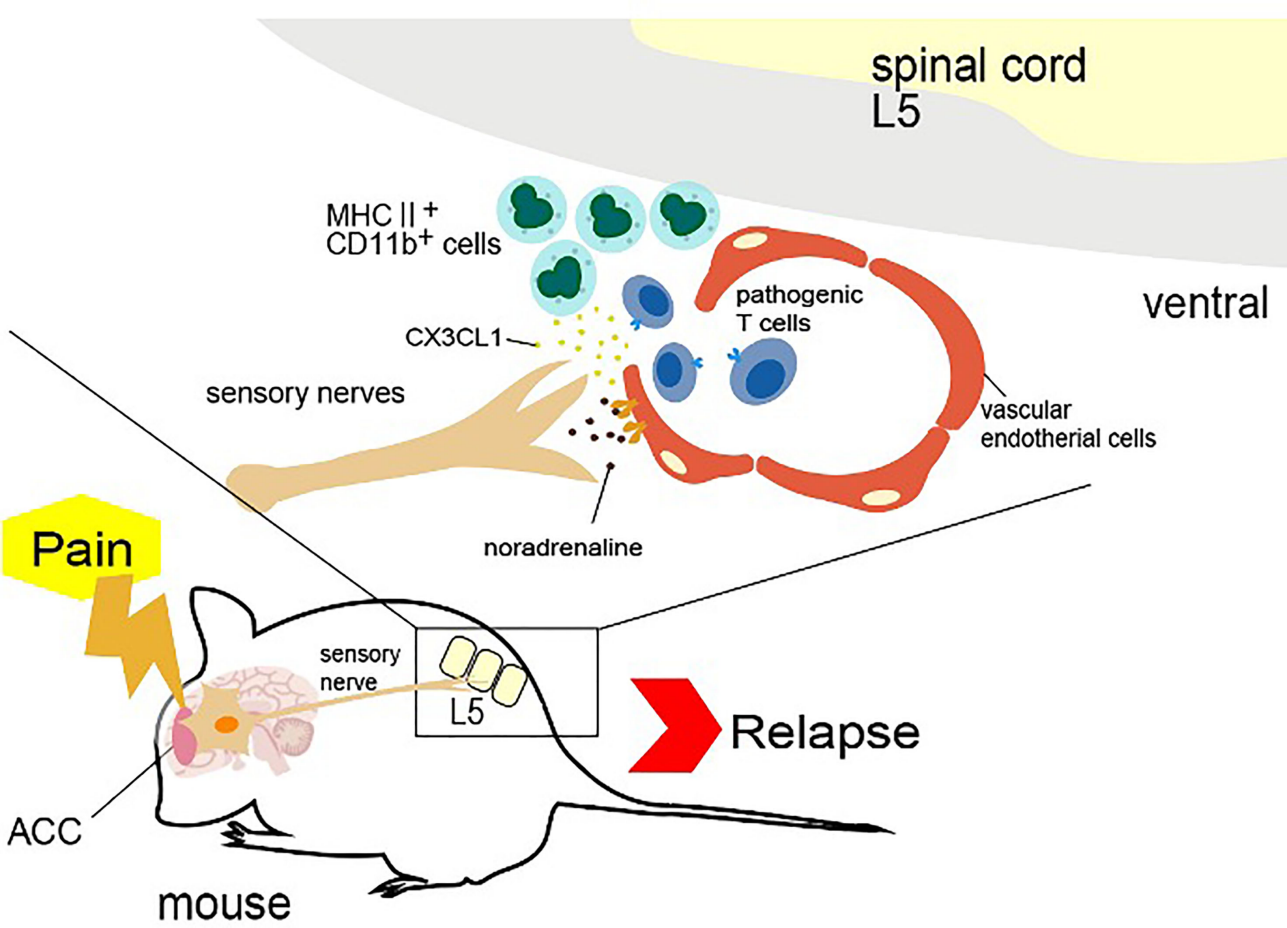
Gravity gateway reflex. Gravity stimulation induces the activation of sensory nerves in the soleus muscles, which connects the fifth lumbar vertebra (L5) dorsal root ganglion, followed by the activation of L5 sympathetic ganglion neurons. Norepinephrine (also known as noradrenaline) from the sympathetic nerves induces the IL-6 amplifier and the infiltration of autoreactive T cells into the spinal cord by upregulating CCL20 at the dorsal vessels of the L5 cord.

Thus, pain induction first causes an accumulation of MHC class II+CD11b+ cells at the ventral vessels of the L5 cord *via* sensory-sympathetic crosstalk. Then, an MHC class II+CD11b+ cell-mediated accumulation and activation of pathogenic CD4+ T cells in the blood occurs, leading to EAE relapse. Because the activated pathogenic CD4+ T cells in the L5 cord express various cytokines, including NFkB and STAT3 stimulators like IL-17, TNFa, and IL-6, the chemokines were induced in the L5 ventral vessels by activation of the IL-6 amplifier, a local chemokine inducer in endothelial cells (44). In other words, pain induction causes sympathetic activation *via* the sensory pathway, which depends on TRPV1 channels, and noradrenaline produced by the sympathetic nerves induces local inflammation in the L5 cord by accumulating immune cells (**Figure 3**).

## Stress Gateway Reflex

Stress is involved in many diseases. We have identified a stress-related nerve circuit that causes gastrointestinal failure and sudden death when pathogenic CD4+ T cells migrate to the CNS from the blood (45). We again employed transfer EAE mice under two stress conditions that have no obviously significant negative effect on the body: light sleep and wet bed environments. Severe gastrointestinal inflammation and heart failure were observed in mice with either stress in the presence of pathogenic CD4+ T cells. A mechanistic analysis showed that the stress stimulation activated noradrenergic neurons in the paraventricular nucleus of the hypothalamus (PVN), which relates to stress and projects to two specific blood vessels surrounded by the third ventricle, dentate gyrus and thalamus, establishing the gateways for the pathogenic CD4+ T cells (45). We named this phenomenon the “stress gateway reflex”. Considering that the dentate gyrus and PVN are stress-related brain regions (46, 47), the stress gateway reflex could play a role even under the steady state. We hypothesized permeability around the specific vessels was increased even in the absence of pathogenic CD4+ T cells, i.e. in the physiological condition that suppresses stress responses, by the activation of neurons in the dorsomedial hypothalamic nucleus (DMH)/anterior hypothalamic nucleus (AHN) (48).

**Figure 4:**
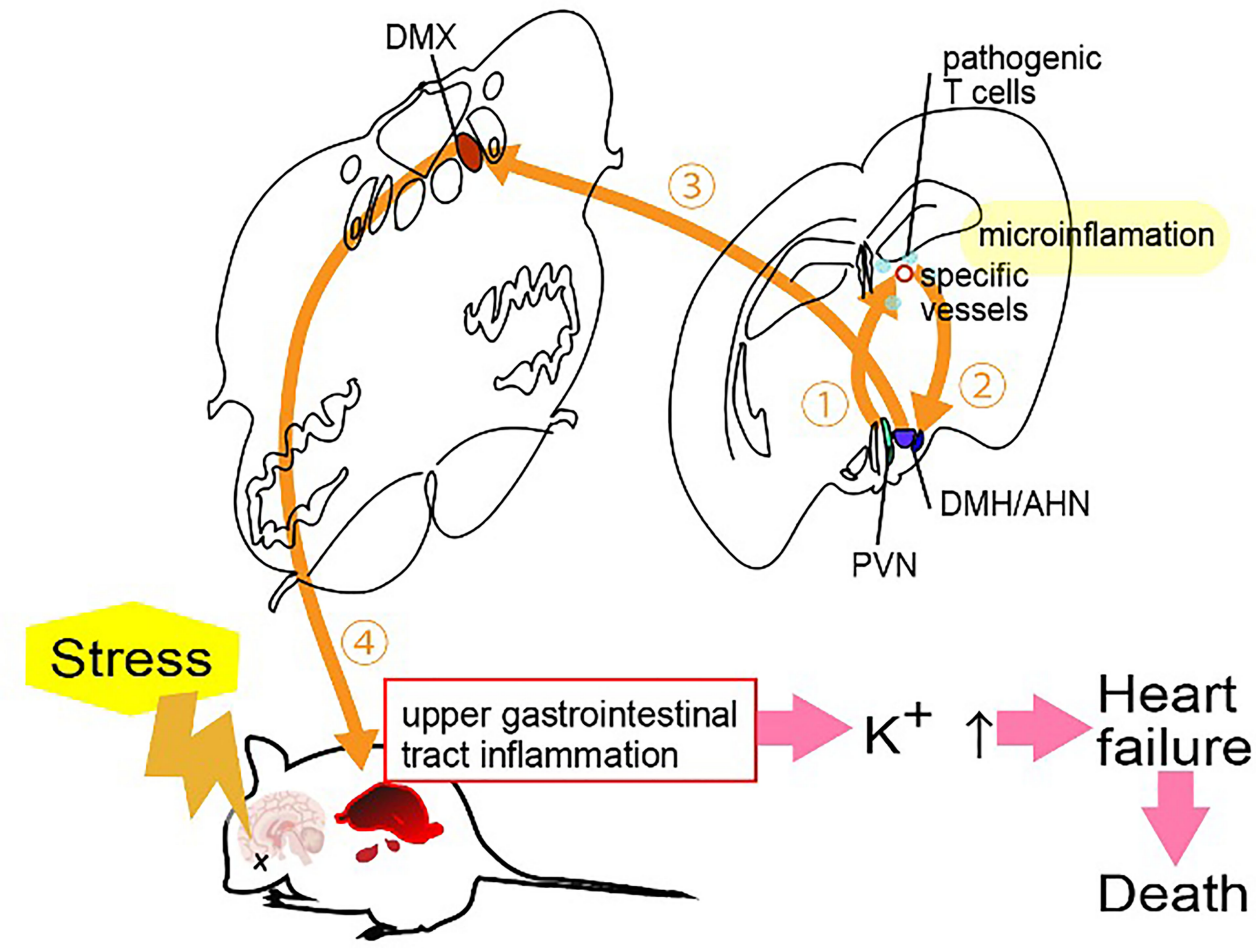
Pain gateway reflex. Pain-induced sensory nerve activation stimulates the anterior cingulate cortex (ACC), which activates specific sympathetic nerves distributed at two ventral vessels of the spinal cord. Because there are many major histocompatibility complex (MHC) class II^+^ monocytes around the L5 cord, norepinephrine secretion at the ventral blood vessels of the fifth lumbar vertebra (L5) cord stimulates the production of CX3CL1 from endothelial cells *via* the IL-6 amplifier. CX3CL1 accumulates MHC class II^+^ monocytes and increases the permeability of blood vessels. Autoreactive T cells in the blood flow accumulate at the vessels to induce experimental autoimmune encephalomyelitis relapse.

On the other hand, during the stress gateway reflex in the presence of pathogenic CD4+ T cells in the blood, PVN-derived noradrenergic neurons upregulate CCL5 expression at the blood vessels in a manner dependent on noradrenaline-mediated NFkB activation, thus recruiting pathogenic CD4+ T cells and MHC class II^+^ monocytes from the blood followed by the activation of pathogenic CD4+ T cells there. Cytokines expressed by activated pathogenic CD4+ T cells accelerated the NFkB activation in endothelial cells and triggered micro-inflammation *via* the IL-6 amplifier (45). Locally, adenosine triphosphate (ATP) produced by the micro-inflammation functioned as a neurotransmitter (49, 50). Indeed, ATP directly activated neurons in the DMH/AHN to activate neurons in the dorsal motor vagal nucleus (DMX) (45). The enhanced activation of the efferent vagus nerve projected to the upper gastrointestinal tract from the DMX to yield severe gastrointestinal inflammation *via* massive acetylcholine secretion followed by hyperkalemia with sudden death (45) (**Figure 4**).

Because the accumulation of immune cells including pathogenic CD4+ T cells at brain-specific vessels should increase the density and pressure between cells followed by an increase of the mechanistic signaling, not only ATP signaling but also mechanical stress activates NFkB in the blood vessels and stimulates the neural pathway to the DMH/AHP.

## Light Gateway Reflex

**Figure 5:**
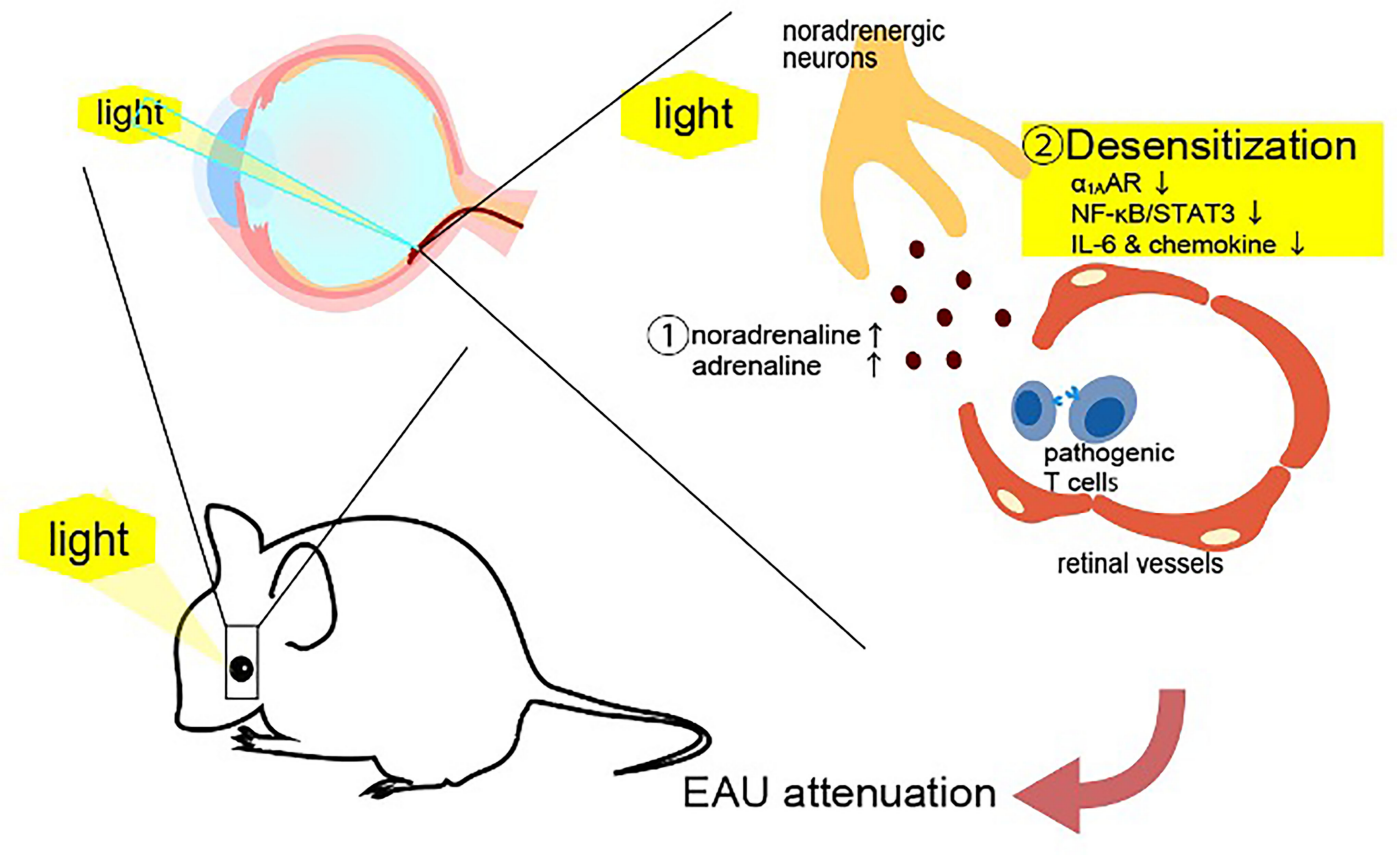
Stress gateway reflex. Chronic stresses including light sleep activate noradrenergic neurons in the paraventricular nucleus (PVN) to establish gateways for immune cells at specific blood vessels in the brain, particularly in the presence of myelin-specific CD4^+^ T cells in the blood. Myelin-specific CD4^+^ T cells accumulate at the specific vessels together with major histocompatibility complex class II^+^ monocytes to develop micro-inflammation there. ATP produced by the micro-inflammation functions as a neurotransmitter to directly activate neurons in the dorsomedial hypothalamic nucleus (DMH)/anterior hypothalamic nucleus (AHN), followed by the activation of neurons of the dorsal motor vagal nucleus (DMX). The resulting activated efferent vagus nerve causes acetylcholine-dependent gastrointestinal inflammation followed by hyperkalemia with heart failure and sudden death.

We showed that photopic light stimulation suppresses the establishment of experimental autoimmune uveroretinitis (EAU), a model of uveroretinitis, in mice (51–53). We noticed a pathological difference between mice kept in photopic light and in mesopic light conditions. A gateway at retinal vascular endothelial cells can be established by NFkB-mediated chemokine expression in retinal endothelial cells to accelerate the infiltration of pathogenic CD4+ T cells against photoreceptors through the blood-retina barrier from the blood (51). EAU mice kept in mesopic light showed more CD4^+^, CD8^+^ and CD11b^+^ cells in the retina than mice kept in photopic light (51). Similar to the other four gateway reflexes, the light gateway reflex includes the activation of sympathetic/noradrenergic neurons in its nerve pathway. Exposure to photopic light downregulated the noradrenergic pathway in retinal vessels by excessive noradrenaline and adrenaline levels from neural terminal in the retina, disrupting the expression of a1A-adrenagic receptor (a _1A_AR) and subsequently suppressing NFkB activation in the retinal vessels (51) (**Figure 5**). The light gateway reflex can be observed not only in uveitis and retinitis, but also in autoimmune intraocular diseases such as retinal vasculitis and ischemic retinopathy. Moreover, activation of the sympathetic pathway can also be observed in autoimmune diseases that present intraocular inflammation such as human leukocyte antigen-B27 (HLA-B27) spondyloarthropathies, sarcoidosis, and juvenile chronic arthritis (54–56). These findings suggest that excessive activation of the sympathetic pathway suppresses the establishment of the gateways for autoreactive T cells in the blood-barrier including in blood-retina one by downregulating adrenergic receptors on the vessels, thus inhibiting tissue-specific inflammation.

Finally, during the pathology of EAU, we noticed an abnormal spatial alignment of neural terminal in the retina due to an accumulation of immune cells and proliferation of retinal nonimmune cells, including fibroblasts. This abnormal alignment likely caused an increase in tension and/or pressure in the retinal lesion. Thus, the mechanosensory pathway affects the pathogenesis of retinal inflammation.

## Concluding Remarks

In summary, we here focused on the gateway reflex, in which mechanical forces, such as gravity, changes the microvascular structure of blood barriers to allow immune cells to invade associated organs, such as the CNS and retina. Inflammation caused by the gateway reflex is different from whole organ inflammation, because autoreactive T cells invade from specific blood vessels in the organs. Pain stimuli induces the accumulation of MHC class II+ CD11b+ myeloid cells at specific ventral vessels of the L5 cord *via* activation of TPRV1 receptor-positive sensory nerves to cause relapse of CNS-inflammation (pain gateway reflex). Although other peripheral-derived CD11b+ myeloid cells in the CNS do not show any obvious function after the pathogenesis, environmental stimuli, including pain sensation, can accumulate them *via* the activation of specific neural circuits by changing the status of specific blood vessels. Regarding, tissue-specific autoimmune diseases, the adaptive immune system, such as T cells and B cells in the blood, plus neural circuits activated by environmental stimulations are critical for the gateway reflex. Autoimmune diseases always commence with the accumulation of immune cells at specific vessels, which increases pressure and tension on the tissue cells as well as the immune cells themselves. Therefore, it is reasonable that mechanotransduction contributes to these diseases. Further elucidation of this transduction as well as immune cells is important for understanding the pathogenesis of various diseases and establishing new treatments.

## Author Contributions

All authors read and discussed the paper. All authors contributed to the article and approved the submitted version.

## Funding

This work was supported by KAKENHI and AMED (MM), the Grant for the Joint Research Program of the Institute for Genetic Medicine, Hokkaido University (MM), the Photo-excitonix Project at Hokkaido University (MM), and the Promotion Project for Young Investigators at Hokkaido University (MM).

## Conflict of Interest

The authors declare that the research was conducted in the absence of any commercial or financial relationships that could be construed as a potential conflict of interest.

## Publisher’s Note

All claims expressed in this article are solely those of the authors and do not necessarily represent those of their affiliated organizations, or those of the publisher, the editors and the reviewers. Any product that may be evaluated in this article, or claim that may be made by its manufacturer, is not guaranteed or endorsed by the publisher.
